# Supplementary material for: Are carbon emissions trading and green financial instruments synergistic? -Comprehensive quantitative research based on content analysis
Source: PLoS One. 2024 Mar 7;19(3):e0298601. doi: 10.1371/journal.pone.0298601 (PMC10919601; doi:10.1371/journal.pone.0298601)
Supplement: S1 Data — (ZIP) [file pone.0298601.s001.zip › Data/地级市Z_F工作报告.zip!/║╙▒▒/└╚╖╗╩╨/└┤╫╘╣1⁄2╓┌║┼í╛┬φ┐╦╩2╛▌═°í┐_2008.docx]

各位代表：

现在，我代表廊坊市人民政府向大会做工作报告，请予审议，并请市政协委员和列席会议的同志提出意见。

过去五年的回顾

过去的五年，是廊坊科学发展、和谐发展的重要时期。在省委、省政府和市委的正确领导下，各级政府和全市人民一道，坚持以邓小平理论和“三个代表”重要思想为指导，全面落实科学发展观，开拓进取，奋发有为，圆满完成了市四届人大一次会议确定的各项目标任务，实现了全市各项事业的新跨越，进入了廊坊加快崛起的新阶段。

——这五年，是廊坊整体实力增强，经济发展质量和综合效益明显提高的五年。

2007年，全市地区生产总值达到883.4亿元，五年翻了一番，年均增长13.6%。全部财政收入达到95.6亿元，五年翻了一番半，年均增长26.4%。财政收入占地区生产总值比重达到10.8%，提高4.4个百分点。全社会固定资产投资达到685.9亿元，增长2.8倍，年均增长30.3%。规模以上工业增加值占地区生产总值比重达到29.1%，提高12.6个百分点。

——这五年，是民生持续改善，人民群众得到实惠的五年。

坚持把解决民生问题作为一条工作主线，通过推进农村“医、学、路、水、保、养”六项工程，解决“群众急需、社会难点、历史欠账”三方面问题，让群众更多地享受改革发展的成果。

一是公共服务和社会保障水平明显提高。新建、改造乡镇卫生院83所，新型农村合作医疗实现全覆盖，51万人次得到补偿。农村免费义务教育全面实现，D级危房全部消除，50万中小学生享受到“两免一补”政策。养老、失业、医疗、生育、工伤五大社保基金规模达到17亿元。住房公积金制度实现全覆盖，累计归集资金24.4亿元。

二是为困难群众织了一张“安全网”。农村最低生活保障基本实现应保尽保。新建、改造敬老院45所，五保户集中供养率达到74.6%。建立残疾人救助基金，为1360户贫困残疾人新建和改造了住房，安排了3802名残疾人就业，13289户符合条件的残疾人家庭全部纳入最低生活保障。建成10所高标准“爱心家园”，全市515名孤儿实现了集中供养，477名有就读能力的孤儿做到了分级就学。我市救助困难群众的做法和经验在全国引起了较大反响。

三是农民生产生活条件得到改善。新建、改造农村公路3123公里，农村公路密度居全省第一，在全省第一批实现村村通油路，在全国率先实现村村通客车。解决了932个村、76万人的饮水安全问题。

四是城乡居民收入增长较快。城镇居民工资性收入增长78.7%。全市城镇居民人均可支配收入达到13975元，农村居民人均纯收入达到5599元，年均分别增长12.5%和7.6%。

——这五年，是立市项目实现突破，产业结构发生重大变化的五年。

通过连续实施“大项目攻坚年”、“招商引资突破年”和“项目质量提高年”，全市上下形成了“全党抓经济，重点抓工业，集中精力上项目”的强大合力。五年累计实施千万元以上项目3572项，完成投资1433亿元。2005年以来争列省重点建设项目263项，占全省16.2%，争列数量连续三年居全省第一。通过省重点项目争取建设用地，累计取得增量指标6万亩，保持全省领先。引进、建设了富士康、华为、中兴、京东方、新奥太阳能电池、首钢(大厂)制造业等一批投资几十亿、上百亿的立市项目，推动我市项目建设进入了新阶段。

园区成为项目建设的主要载体。省级园区由4个增加到10个，总数居全省第一，每个县(市)都有了省级园区。建设了一批工业新区和乡镇产业功能区。国家级出口加工区封关运行。

主导产业发展壮大。高新技术产业迅速扩张，增加值年均增长25%以上，达到85.4亿元，占地区生产总值比重达到9.7%。其中，电子信息产业基地有了雏形，销售收入年均增长70%以上，达到59.5亿元。会展旅游产业方兴未艾，打造了“5·18”国际商务节、“9·26”中国农产品交易会、中国国际电梯展和碧海渔具展等一批会展品牌。

——这五年，是基础设施进一步改善，环境优势日益强化的五年。

基础设施方面，五年投入200多亿元，完成了路网建设、输变电线路改造、通讯扩容、水利设施治理等一批基础设施工程。建成了11条干线公路，谋划启动了3条高速公路建设。建成投运110千伏以上变电站19座、输变电线路596公里，电力保障能力明显增强。南水北调配套工程奠基。

软环境方面，金融创新、信用体系、人才高地等项工作取得新成果。信贷规模迅速壮大，2007年新增贷款增速全省第一、增量全省第二，我市被评为中国最具魅力金融生态城市。建成企业和个人信用数据库，开通廊坊信用网，信用体系建设迈出实质性步伐。全市人才密度指数达到13.79%，五年提高3.72个百分点。

——这五年，是农村经济蓬勃发展，新农村建设成果丰硕的五年。

全面落实各项惠农政策，取消了农业税，人均减少税费负担123元。累计发放粮食直补、良种补贴、农机具购置补贴等3.8亿元，惠及293万农民，人均增收278元。粮食连续五年丰收，畜牧、蔬菜、林果花木三大产业占农业总产值比重达到80%。农业产业化经营率达到61%，省级以上龙头企业发展到20家。完成城乡造林绿化182万亩，植树1.08亿株，相当于上一个五年的4.5和5.5倍。新建农村沼气池4.52万个。林业病虫害、有害生物防治和禽流感等重大动物疫情得到有效控制。建成文明生态村300个。

——这五年，是城市面貌大变样，城市品牌越来越响亮的五年。

城建“十大工程”扎实推进。建成博物馆、图书馆、摔拳跆中心、全民健身中心、体育训练中心等一批文体设施，实施了综合市场建设、人民公园改造、环城水系、市区道路和郊区快速路等一批工程。城市环境不断改善。市区绿化覆盖率达到46%，人均公共绿地12.2平方米，空气质量二级以上天数连年保持在330天以上。在全省率先采用数字化城市管理模式，开展“大拆违”、“大整治”，清理了长期积存的违法临建，城市管理市民满意度明显提高。

城市知名度和影响力逐渐提升。荣获中国人居环境奖，跻身中国优秀旅游城市、国家环保模范城市、国家节水城市、国家园林城市、全国绿化模范城市和全国科技进步先进城市行列，成为ISO14000国家示范区和国家可持续发展实验区。

——这五年，是各项事业长足发展，经济社会协调并进的五年。

科技创新体系逐步完善，被科技部批准为“国家信息产业基地”，成为全省首批“创新型城市试点”，市生产力促进中心成为全省首家国家级示范中心。教育事业蓬勃发展，在全省率先基本普及高中阶段教育，首批普及学前三年教育。文化体育事业走向繁荣，“文化信息资源共享工程”跨入全国先进行列，30余件艺术作品获国家级奖项，连续在十一、十二届省运会创历史最好成绩，承办了全国马拉松冠军赛等多项国家级赛事。食品药品放心工程深入实施，人民群众饮食用药安全有了保障。公共卫生体系建设全面推进，重大传染病预防控制成效明显。人口与计划生育工作得到加强，低生育水平保持稳定，利益导向政策体系日趋健全，人口出生性别比例偏高问题开始解决。“蓝天、碧水、净土、绿色、宁静”五大工程推向纵深，连续8年成为省环保目标考核优秀市，跻身全国7家“保护臭氧层示范省市”行列。

五年来，各级政府的职能不断转变，社会管理和公共服务切实加强。

行政审批制度改革加快，五年共削减审批事项965项、收费项目213项，建成全市行政审批服务中心，46个市直部门和办事机构的293项审批服务事项进入大厅，按时办结率和市民满意度分别达到99.7%和99.8%。行政效能显著增强，开通了行政权力公开透明运行网，广泛开展行风评议，大力实施效能监察，廉政建设逐步向纵深展开。国有资产管理体制和国有企业改革稳步推进，监管效能明显提升。安全生产工作不断强化，五年中事故起数、死亡人数、直接经济损失年均减少30%、18%和11%。社会稳定进一步巩固，信访“十百千”工程效果明显，“平安廊坊”建设扎实推进。特别是在这五年中，我们取得了抗击非典的重大胜利，妥善解决了东方大学城遗留问题和国有企业改制等重大问题，有力地维护和保持了全市安定团结、风清气正的好局面。

刚刚过去的2007年是四届政府任期的最后一年。在起点提高、约束增多、竞争加剧的情况下，我市主要发展指标超出预期，地区生产总值和全部财政收入比上年分别增长15%和40.4%，一般预算收入增速自分税制以来首次超过全部收入增速。一年中，启动实施了一批事关长远的战略项目。投资71.1亿元的廊沧高速公路奠基动工，投资18.5亿元的密涿支线高速公路、投资49亿元的京津南通道高速公路廊坊段前期工作进展顺利。万庄生态新城建设启动，龙河中心区完成规划设计，中科廊坊科技谷成为“联合国高科技产业暨国际合作示范项目”。着眼未来区域发展，谋划了“一带两基地”，即，北部潮白高新技术产业带，中部永定信息产业基地和南部现代制造业基地。此外，全民创业、民生改善、社会建设等方面也创造了新业绩。

各位代表！

过去的五年，是廊坊经济社会发展实现历史性跨越的五年，人民群众比较充分地享受到了发展的成果，廊坊加快崛起的发展态势和美好前景已经展现开来。这些成就的取得，是省委、省政府和市委正确领导的结果，凝结着全市人民团结奋斗的智慧和汗水。在此，我代表廊坊市人民政府，向全市干部群众，向各民主党派、人民团体和各界人士，向人大代表、政协委员，向驻廊人民解放军、武警官兵和公安干警，向驻廊中省直单位，向关心、支持廊坊发展的海内外朋友，致以崇高的敬意和诚挚的感谢！

奋斗的历程难以忘怀，发展的经验弥足珍贵。回首五年，我们深刻体会到：

一是必须坚持走符合廊坊市情的科学发展道路。廊坊最大的市情是区位，最明显的优势是环境，最突出的问题是实力不强。推动廊坊发展必须深刻把握市情，靠环境建设把区位条件转化为发展优势，靠项目带动把实力做大做强，靠经济社会良性互动实现可持续发展。

二是必须坚持创新理念和方法。不断解放思想，坚持规划先行，用好市场机制，善于重点突破，解决好“桥”和“船”的问题，确保目标任务实现。

三是必须坚持一切为了人民。把执政为民作为政府的崇高使命，把人民的小事当作政府的大事，从解决群众最关心、最直接、最现实的问题入手，尽心尽力、扎扎实实地向前推，让廊坊人民越来越有幸福感。

四是必须坚持一切依靠人民。廊坊人民热爱家乡、建设家乡的豪迈激情和冲天干劲，是我们加快发展的最大力量；广大群众的理解和支持，是我们战胜一切艰难险阻的制胜法宝。

五是必须坚持务实争先。为了廊坊的发展，全市干部群众始终保持一种激情奉献、拼搏进取的精神，始终保持一种奋发向上、生机勃勃的朝气，始终保持一种敢为天下先和严细深实快的作风。务实争先已经成为全市上下的主旋律。

在肯定成绩的同时，我们也清醒地看到，当前经济社会发展和政府工作中还存在不少问题，也面临许多挑战。主要表现为：经济总量仍然偏小，综合实力还不强；主导产业规模不大，传统产业比重偏高，服务业发展滞后；相对于日益增长的民生需求，我们的许多工作还没有跟上；部分干部的思想不够解放，政府职能转变还不够到位，行政效能和公务员素质有待提高，廉政建设需要加强，等等。此外，土地、资金、环境等约束不断增强，防止经济过热和通货膨胀的压力不断加大，前进的道路并不平坦。

与此同时，我们更应看到未来发展的难得机遇。环渤海尤其是京津冀地区正在成为中国新的增长极，有利于我们把区位条件加速转化为发展优势。实践表明，每一次宏观调控都为廊坊加速发展提供了契机，今年的宏观调控，也将有利于我们沿着既定的科学发展之路实现加快崛起。近些年来，在历届党委、政府和全市人民的共同努力下，我们已经有了比较坚实的发展基础，形成了戮力同心的发展局面，具备了加快崛起的发展条件，省委、省政府对廊坊寄予厚望。只要我们认清形势，顺势而为，解放思想，埋头苦干，就一定能把廊坊的事情办得更好！

2008年的目标任务和重点工作

2008年是新一届政府的届首之年。做好2008年的工作意义重大。

政府工作的总体要求是：高举中国特色社会主义伟大旗帜，以邓小平理论和“三个代表”重要思想为指导，深入贯彻落实科学发展观，遵循“依法、科学、民主、文明”的工作方针，更加注重培育主导产业，更加注重全民创业，更加注重环境建设，更加注重民生，更加注重社会和谐稳定，努力实现“科学发展、进位争先、和谐安定、富民强市”。

综合考虑多方面因素，2008年全市经济社会发展主要预期目标是：地区生产总值增长15%左右，地方一般预算收入增长30%以上，城镇固定资产投资增长35%以上，城镇居民人均可支配收入和农村居民人均纯收入分别增长10%和7%以上，城镇登记失业率控制在3.5%以内，万元GDP能耗下降4.5%，二氧化硫、化学需氧量排放在2005年基础上分别下降5.5%和6.5%。

做好今年工作，关键是突出“两条主线”，把握“两大抓手”，做好“九方面工作”。

“两条主线”，就是壮大经济实力和解决民生问题。“两大抓手”，就是一手抓招商引资上项目，一手抓全民创业增主体。 “九方面工作”，包括项目、园区、全民创业、现代服务业、新农村建设、城市建设、可持续发展、改善民生和政府自身建设，等等。

一、推进重大项目建设，积极培育产业集群

深入实施“项目质量提高年”，以推进重大项目建设为抓手，引龙头，抓配套，建基地，加快培育新兴主导产业。

抓好十大龙头项目。确保在建项目顺利建设，促进后续项目落地。富士康项目，促进一期工程尽快投产达效，力争引进20家配套厂商进驻，协调解决好二期工程征地、拆迁，尽快形成基地框架，加快龙河高新区配套设施建设。华为项目，加强投产前各项服务，确保三季度陆续投产运营。新奥太阳能电池项目，一期工程9月底前竣工投产。中兴项目，建成一期光通讯及光传输设备、软件研发等项目主体厂房，加快二期项目前期进度。首钢(大厂)制造业项目，年内完成投资20亿元的首钢机电公司搬迁。中科廊坊科技谷项目，完成投资30亿元，一批孵化企业进入创业大厦，确保中科院工程热物理研究所中试基地、创业孵化中心等4个项目如期开工。万庄生态新城项目，加快起步区建设，及早实现区内道路通车，推进生态核修复、五星级酒店、永旺国际购物中心等项目建设。南玻玻璃项目，完成投资11亿元，确保10月份投产。燕达医院项目，投资10亿元，完成老年养护中心一期、医学科学研究院及医院主体工程。成功大广场项目，完成投资8亿元，开工面积30万平方米，年底部分工程结构封顶。

谋划新上一批重大项目。着眼于构筑集群优势，围绕壮大电子信息、生物医药、汽车摩托车零部件、家具制造、会展旅游等主导产业，面向世界500强、中央直属大企业和全国民营百强企业，积极转变招商方式，谋划引进一批龙头项目。按照产业规划，以集聚发展为目标，以京津为依托，靠园区载体和高效服务招商。进一步发挥民营骨干企业家的作用，鼓励他们多上项目多投资，为家乡发展多出力。做好省重点项目和重点产业支撑项目争列工作，确保重点项目个数和土地增量指标在全省领先。全年谋划实施亿元以上项目300个，总投资1100亿元。

推进产业基地建设。一是确保信息产业基地建设实现跨越。深入做好国内百强电子信息企业招商，争取再引进一批大项目和配套项目，把大项目发展成大基地。根据落地大项目在文化、健康、教育等行业的需要，因势利导，做好服务，在更多领域发挥其对地方经济的拉动作用。二是力争生物医药产业基地建设实现突破。按照信息产业基地的建设模式，进一步完善产业规划，在引进重大项目上下功夫，年内建设3个生物医药专业园区，承办好第三届中国医药工业百强企业年会。通过努力，把生物医药产业发展成廊坊第二大高科技产业。

二、增强园区聚集效应，打造经济发展增长极

立足于资源集约、企业集聚、产业集群，不断提升园区水平与档次，使园区真正成为项目工作的主战场、区域经济的增长极。

完善园区体系。围绕主导产业，重点打造10个省级园区，增强对项目的吸附力和承载力。在省级园区中谋划发展一批“园中园”，启动建设一批新的园区，在重点镇谋划建设一批产业功能区。制定园区发展总体规划，结合村庄整理，推进地域相近、产业互补的工业园区协调发展、共同提高。

强化园区功能。着眼园区功能和园区水平提升，加快实现“五有”目标。一有高水平规划，按照“一县、一园区、一产业、一新城”的思路，对每个园区的规划进行整体提升；二有战略投资者，建立和完善政府主导、企业运营、市场化运作的建设机制；三有专业招商队伍，选择引进一批有活力、有经验、有水平的招商人才；四有招商中心，提供规划展示、招商洽谈、商务接待等各项服务；五有完善的基础设施，全面加强路、水、电、气，特别是污水、垃圾处理等基础设施建设。

加快机制创新。创新服务机制，大力发展中介服务机构，为投资者提供管理、人才、法律、审计等各类专业化服务。创新督导机制，成立园区工作领导小组，负责全市园区的规划、指导与协调。创新考核机制，出台园区考核评价办法，对各园区实施分类考核、动态管理。

三、激活市场主体，推动全民创业和民营经济发展

项目强市，创业富民。加快崛起的根本动力在于全民创业，有效途径在于激活创业主体。我们的目标是，让人民群众的创业热情竞相迸发，让民营经济成为最有力的支撑，让创业成为廊坊的一种文化。主要抓好“四四五工程”。

实现“四个一目标”。争取平均每个县新增民营经济单位1000个，全市新增10000个以上；平均每个县抓100个重点村，全市抓1000个。力争千人民营企业个数达到5.5户。

实施“四个带动”。一是富村带动，继续筛选创业基础好的村，带动穷村弱村。二是企业带动，鼓励大户企业帮扶村集体和家庭开展创业。三是梯队带动，排出实力强的大企业，实力较强的中等企业，以及成长性好的中小企业，形成梯队结构，实施分类扶持，促进企业发展壮大。四是领导带动，落实好领导包村“十百千”工程，抓好创业重点村。

强化“五个支撑”。一是政策支撑。出台鼓励措施，放宽创业的经营范围、注册资本、证照限制等，制定费用免除等具体办法。二是行政支撑。推广开放式政企对接“超市”、餐会等活动，畅通政企沟通的“绿色通道”；针对吃拿卡要、办事低效等问题，加强明查暗访，广开监督渠道，探索服务中小企业的效能监察新模式；加强调度考核，将全民创业纳入县(市、区)考核体系。三是人才支撑。免费组织企业家进修，对中小企业职工实施远程教育培训，力争培训18万人次以上。四是金融支撑。抓住国家大力推进农村金融体制改革的契机，积极发展小额贷款公司、村镇银行、担保公司、农村资金互助合作组织等新型农村金融机构，为全民创业提供较为充裕的资本支持。五是载体支撑。规范提升现有创业辅导基地，每个县(市、区)新建一个高标准的创业辅导基地。

四、加快发展现代服务业，促进产业结构优化升级

面向域内和京津两大市场，着眼生产生活两方面需求，在改造提升传统服务业的同时，以三河、大厂、香河、永清、固安和廊坊市区为依托，以会展旅游、现代物流、健康服务等行业为重点，集中力量发展现代服务业，建设环北京现代服务产业带。

壮大会展旅游业。对廊坊会展中心等会展设施进行改造提升，完善场馆功能和配套服务体系，提高承接国际大型展会能力。继续办好“5?18”国际商务节、“9?26”中国农产品交易会、中国热气球节、碧海渔具展、中国国际电梯展等大型会展活动，提升专业层次，打造更多的品牌展会。加快建设霸州“温泉城”、永清“农家游”、三河燕龙马术俱乐部、金丰农科园等旅游精品项目。

加快发展现代物流业。依托广阳物流中心，谋划建设“内陆无水港”。推进三河汇金陆港物流、廊坊宝湾物流、胜芳产地物流、杨芬港津港物流等重点项目建设。依托香河家具城、胜芳国际家具博览城等资源，发展专业性区域物流。

培育金融服务业。依托京津，重点发展金融后台服务和金融休闲商务，谋划建立数据中心、清算中心、研发中心、呼叫中心。积极引进域外金融机构，鼓励金融业态创新，发展金融租赁、创业投资、基金管理、风险投资和融资担保等业务，培育多层次的金融市场体系。依托中科廊坊科技谷，推动软银、摩根士丹利等风险投资公司尽快落户。

打造健康服务业。聘请权威专业机构编制产业发展规划，制定具体招商引资计划，确保项目引进实现突破。依靠龙头带动，重点发展“一站式”健康体检中心、疗养机构和医药商业物流企业，积极发展健康教育、健康规划、健康咨询等相关产业。

推进信息服务业。推动嵌入式软件、系统软件和应用系统开发服务业的发展。围绕本地电子信息龙头企业，大力引进配套软件企业。加快廊坊开发区和燕郊开发区服务外包产业基地建设。

支持文化创意产业。制定发展规划，出台扶持政策，着眼京津产业调整和市场空间，发展广告传媒、创意设计、数字娱乐等产业。谋划文化创意产业园区，建设万庄创意产业基地，吸引一批知名公司和专业人才，促进产业集聚。

五、扎实推进新农村建设，统筹城乡协调发展

推进农业产业化和农村重点工程，创新农村工作机制，提高农业综合生产能力，促进农民增收和农村财富积累。

开展村庄整理。着眼城乡一体化和新农村建设，进行村镇布局调整，推进村庄整理。对全市3222个行政村统一规划，按照“转变一批、提升一批、改善一批、迁建一批”的思路，遵循“规划先行、政策引导、村民自愿、多元投入”的原则，筛选100个基础较好的村作为试点，取得经验，分步推进。

加快农业产业化进程。以省“111行动”为抓手，重点扶持20个市级种养基地，争取新增5-6家省级重点龙头企业；力争1-2家企业入选省级十大品牌企业，新增1-2个全省加工示范基地县、1-2个中国驰名商标。发展农民专业合作经济组织200个以上。农业产业化经营率达到62%以上。

创新四个工作机制。一是创新农村劳动力就业机制。探索建立市、县、乡、村四级就业培训服务体系，建立劳动力网络台帐，整合全市培训资源，开展定向培训、订单服务，推动用工企业与农民工建立稳定的劳动关系。二是创新集体经营积累机制。筛选100个村，进行农村集体资产管理试点，探索建立有效的资产经营积累模式，促进农村公共设施的可持续发展。三是创新农村财务委托代理机制，促进农村财务管理再上新台阶。四是创新土地流转机制，积极引导土地向种养大户和农业企业集中，提高土地集约化水平。

推进六项重点工程。一是文明生态村工程，新建文明生态村100个。二是生态家园富民工程，新建沼气池1.6万个，建立“沼气、沼渣、沼液综合利用示范区”1-2万亩，切实加强农村环境保护。三是农村信息化工程，重点推广农业专家在线、农业短信等8种服务。四是“百万吨奶”工程，力争奶牛存栏达到13万头，产奶40万吨。五是“一村两员”工程，每个村培养1名农业技术员和1名动植物协防员。六是“一十百千”绿化工程，建设“一个森林城市”，打造“十条国省干道精品绿廊”，推进“百镇环城林建设”，完善“千个农田林网方格”。全年造林绿化30万亩，义务植树600万株。

六、提高城市规划建设管理水平，增强城市竞争力

以城乡总体规划为龙头，启动新的“三年大变样”工程，推进城市管理的科学化和现代化，提升城市品位，不断增强中心城市的辐射带动作用。

加强中心城市组团规划和管理。“小三点”之间进一步完善规划，真正体现连之以路、隔之以绿的组团格局。“大三点”之间重点加强对基础设施规划、控制性详细规划、主要道路景观规划、厂区规划的管理和控制，保持各组团规划的协调一致。及早谋划启动“大三点”之间的快速通道。

推进四个旧城区片和城中村改造。包括启动火车站、三角地、尖塔、龙河中心区区片改造，启动董村、西户屯、苗场、祖各庄、北史家务及龙河高新区、万庄生态新城的城中村改造，力争三年内市规划区60个城中村改造工程全面展开。

提升中心城市整体功能。一是改造市区西、南、北三个出入口。重点抓好道路的拓宽、绿化和亮化。二是实施雨污分流改造。新建污水处理厂两座，改造24公里雨污分流管道，实现“污水管中走、清水渠中流”。三是加强环境卫生建设。采用BOT形式，启动垃圾发电项目。四是开展城市交通便民工程。改造7个市区主要干道交叉口，增加公交车辆和线路。五是实施绿化精品工程。重点抓好水系景观、快速路景观、道路提升等8项工程，打造以环城绿化为骨架、道路绿化为网络、水系绿化为灵魂、游园绿化为亮点的绿化格局。在此基础上，谋划启动新的“三年大变样”工程。用三年时间，围绕城市经济、城市平台、民生保障、生态环保四个方面，实施城区拓展、村庄整理、交通畅通、水系生态绿化等10项重点工程、共120个子项目，估算总投资近千亿元，实现城市面貌“每年一大步、三年大变样”。

提高城市管理水平。全力争创全国文明城市，并以此为契机，推动城市管理上台阶。深化城区万米网格化精细管理，整合部门管理职能，管理与监督职权相分离，建立为群众服务的协同联动机制，实现由粗放管理向集约管理和精确管理转变。进一步建立和完善教育、卫生、文化等社区服务体系，扎实开展“星级社区创建评定活动”，不断提升社区管理服务整体水平。

七、进一步优化环境，增强可持续发展能力

以破解发展制约因素为着力点，推动体制机制创新，全面加强基础工作，优化基础环境。

推动城乡统一规划布局。以落实《城乡规划法》为契机，坚持高起点、高标准，制定完善全市国土整体规划、城乡建设体系规划、基础设施建设规划和第三产业发展规划等。对全市6429平方公里土地进行统一安排，对主要交通干线两侧、重要地段的土地实行完全控制，严防随意开发。强化“规划即法、执法如山”意识，确保规划的强制性，既不能为发展设置障碍、留下隐患，更要给加快崛起创造和提供充分必要的条件。

加快构建北、中、南三大板块。以“一带两基地”建设为核心，加快北、中、南三大板块发展。北部，积极推进北三县经济一体化，成立北三县实业开发有限公司，开发建设潮白高新技术产业带。中部，依托廊坊开发区、龙河高新区、固安工业区、永清工业区及万庄生态新城，打造永定信息产业基地，努力建成全国最具影响力的信息产业聚集区之一。南部，以霸州、文安、大城现有工业为基础，通过升级改造，打造现代制造业基地。

着力实施战略性基础设施工程。加快廊沧、密涿支线、京津南通道廊坊段等高速公路及连接线建设，加快廊涿高速公路九州、永清、固安三条连接线和唐通线等干线公路建设，加快农村公路改造，努力形成连接城乡、四通八达的公路交通网络，到“十一五”末，实现“县县通高速、县城通一级路、乡镇通二级路”。加快推进南水北调工程廊坊干渠及广阳水库建设。切实做好IGCC电厂和北京新机场的争取工作。

努力缓解资金瓶颈。针对银根紧缩新形势，千方百计增强融资能力，努力实现“四个突破”。一是进一步优化金融生态环境，在吸引域外商业银行、股份制银行等各类金融机构和战略投资者上求突破。二是规范中小企业透明运行机制，积极推行联保贷款，大力发展担保机构，在中小企业融资上求突破。三是进一步完善金融评价奖励办法，扩大承兑汇票等金融产品应用，在金融产品创新上求突破。四是创新对话沟通机制，在政金企紧密合作上求突破。与此同时，充分利用多层次资本市场，面向主板和创业板，大力推动梅花味精、欧克化工等企业上市，促进企业发行债券。

大力完善信用体系。加快推进信用信息平台建设，借助政府公务网实现信息共享。按照《推广应用信用产品的实施意见》，在土地使用权出让、建筑市场招投标、政府采购等重点领域应用信用产品，形成企业失信惩戒机制。出台《个人信用信息征集使用办法》，完成“一卡通”系统建设，启动个人账户、信贷、外汇、缴税、公共信息子系统建设。

积极推进改革创新。推进国有企业公司制、股份制改革，做好国有产权、股权转让工作，加强国有资产运行风险管理。推进医疗卫生事业体制改革，建立公共卫生、农村卫生、社区卫生合作机制，构建城乡基本医疗服务网，对市中医院、市妇幼保健中心进行改制。推进强镇扩权，选择经济实力较强的镇作试点，扩大镇级政府权限，充分发挥重点城镇对区域经济及全市经济社会发展的带动示范作用。

强化生态环境优势。生态环境是廊坊的核心竞争力，必须倍加珍惜。我们将以更高的工作标准和更大的工作力度，扎实推进环保和生态环境建设。城区空气质量二级以上天数保持330天以上，集中式饮用水水质达标率达到100%，工业废水排放达标率100%，城市污水处理率80%以上。主要做好四项工作：一是推进生态市建设。落实《廊坊市生态市建设规划》，三河和霸州建成省级环保模范城市。二是狠抓综合整治。抓好“三个一批”，淘汰一批污染严重、高耗能、高耗水的企业；限期治理一批排放不达标的企业，到期仍不达标的吊销营业执照；挂牌督办一批轧钢、造纸、化工、建材、制药等违法排污企业。文安和霸州确保完成省下达的“双三十”节能减排目标任务。三是继续实施“1225”工程。确定并实施100项减排工程，重点监控200家企业，加强对20个园区和乡镇年度减排工作的考核，三年内关停取缔500家重污染企业，今年完成100家。四是增强节能节水意识。鼓励应用各类节能技术，发展节能建筑，推广使用太阳能等可再生能源。推动水资源的可持续利用，加大地下水资源保护力度，科学适度开发地热资源。

八、更加重视民生，加强和谐社会建设

深入实施农村“六项工程”，进一步解决好民生“三方面问题”，全面推进社会建设，切实维护社会稳定，增进经济社会发展的和谐程度。

进一步解决“三方面问题”。在扩面提质、制定标准、完善机制上下功夫，不断拓展实施的广度和深度。

“群众急需”方面：加快农村中小学布局调整、危房改造和标准化学校建设，两年解决取暖无保证问题，三年建成100所农村标准化初中和小学。加快廊坊市第十四小学和第一实验小学的扩建工程，逐步解决市区小学班容量大的问题。建立孤儿救助长效机制。提高和巩固五保户供养水平。三年解决城市低收入家庭住房困难问题。保持城镇“零就业家庭”动态归零。扩大“新农合”受益面和受益程度。启动城镇居民基本医疗保险试点工作，积极探索城乡居民大病救助和保险的有效办法。继续推进联村集中供水工程，续建、新建9座水厂，解决12万人的饮水安全问题。

“社会难点”方面：在全市建立食品药品监督网，村街建立食品安全监管组织，保证群众食品药品安全。狠抓一批盗窃、破坏电力电信设施的重大积案。推进旧小区综合改造，提升旧小区物业管理水平和小区环境。启动长途汽车站搬迁工程。

“历史欠账”方面：出台《城市社区卫生工作发展设置规划》，实施“社区卫生服务形象工程”，加强社区卫生服务机构建设和管理。加强优抚对象救助工作，重点解决好优抚对象的住房和就医问题，新建、改造7所光荣院。探索建立农村社会养老保险制度，推进新型农村养老保险试点。加强公共文化体系建设，丰富农民文化生活。改造农村公路350公里，维修改建25座闸桥涵。

扎实推进社会建设。注重科技创新，优化创新环境，着力引进科研院所，建立一批工程技术研发中心和行业生产力促进中心，推进中国发明家成果产业化基地建设，争创国家级科技企业孵化器和国家特色产业基地。深入推进“人才高地”建设，面向支柱产业、新上项目、科技园区和非公企业，创新项目人才对接机制，启动产业人才开发计划，加大人才引进力度，全市人才密度达到14.86%。加快教育事业发展，在提高义务教育水平、巩固学前教育和高中阶段教育基本普及成果的同时，增加各级财政投入，大力发展职业教育，组建河北省(廊坊)电子信息职业教育集团。探索多种形式的办学模式，出台扶持政策，提升民办教育规模和水平。完善公共文化服务体系，加强市、县、乡、村文化设施建设，开展丰富多彩的群众文化活动，加强文化资源的保护利用，推进廊坊大剧院建设。加大广播电视事业投入，提高农村覆盖水平，增强广播电视的服务功能。加快体育场馆等基础设施建设，筹备好第十三届省运会。建立市医疗急救指挥调度中心。完善计划生育利益导向政策体系，稳定低生育水平，人口自然增长率控制在5.92‰以内。通过落实政策、创新机制等多种途径，力争城乡居民收入包括工资收入明显提高。认真落实各项少数民族和宗教政策。支持大厂争创全国少数民族科学发展示范区。

切实维护社会稳定。做好北京奥运会期间的安全保卫工作。搞好矛盾纠纷排查化解，对城镇拆迁、土地征用、劳动保障等群众反映相对集中的问题，专题研究，逐项解决。加强“三位一体”调解体系建设，把矛盾纠纷化解在萌芽状态。建立健全隐患排查治理机制和安全投入保障机制，建设重大危险源监控预警系统，抓好安全生产应急救援体系建设，严防重大事故发生，保持安全生产指标全省最低。强化应急处理机制，提高对重大灾害的预警、评估和防范处理水平。深入开展“平安廊坊”建设，保持安全稳定的社会局面。提高国防动员能力，加强“双拥”和国防后备力量建设。

九、加强政府自身建设，努力提升工作水平

牢记立党为公、执政为民的宗旨，适应新形势新要求，我们必须以解放思想为动力，以提高政府执行力和公信力为重点，大力推进政府自身建设，切实做到为民、务实、清廉。

提高执政能力。创新政府管理理念、管理机制和管理方式，不断完善决策、执行、监督运行体系。坚持科学民主决策，完善公众参与、专家论证、政府决策相结合的机制，对涉及经济社会发展的重大事项，充分论证评估，力求科学缜密，充分反映民意。坚持创新工作方法，善于抓主要矛盾，突破关键环节。坚持监督检查，健全岗位责任制、重点工作责任制和行政问责制，深化机关效能和绩效评估，对目标责任执行情况跟踪问效、考核问责，促进工作提质提速。

推进依法行政。全面落实依法行政实施纲要，增强公务员依法办事意识。加强市、县两级行政服务中心建设，进一步削减行政审批事项和收费项目。健全行政执法责任制和执法过错追究制，对各类行政处罚权实施相对集中，从源头上防止多头执法、重复执法、交叉执法，规范行政执法自由裁量权。

保持清正廉洁。健全和完善教育、制度、监督并重的预防和惩治腐败体系，落实“一岗双责”制度，加大从源头预防和惩治腐败的力度。继续开展行风评议，推动政府各部门改进工作，提高服务质量和办事效率。弘扬艰苦奋斗的优良传统，反对铺张浪费，集中财力办大事。坚决执行廉洁自律的各项规定，提高政府工作透明度，让权力在阳光下运行。

加强作风建设。大兴求真务实之风，多干打基础、利长远的事，多办群众拍手叫好的事，努力创造经得起实践和历史检验的政绩。注重公务员队伍建设，加强学习培训，完善绩效考核制度，努力提高履行职责、服务群众的本领。

各位代表！

回顾五年令人鼓舞，展望未来催人奋进。廊坊已经站在新的历史起跑线上。我们肩负着人民的期盼，踏上了加快崛起的征程。让我们高举中国特色社会主义伟大旗帜，全面贯彻党的十七大精神，深入落实科学发展观，在省委、省政府和市委的坚强领导下，振奋精神，奋力拼搏，为创造廊坊更加美好的未来而不懈奋斗！
